# Chemotherapeutic and Safety Profile of a Fraction from *Mimosa caesalpiniifolia* Stem Bark

**DOI:** 10.1155/2021/9031975

**Published:** 2021-12-07

**Authors:** Paulo Michel Pinheiro Ferreira, Renata Rosado Drumond, Jurandy do Nascimento Silva, Ian Jhemes Oliveira Sousa, Marcus Vinicius Oliveira Barros de Alencar, Ana Maria Oliveira Ferreira da Mata, Nayana Bruna Nery Monção, Antonia Maria das Graças Lopes Citó, Ana Fontenele Urano Carvalho, Davi Felipe Farias, Patrícia Marçal da Costa, Adriana Maria Viana Nunes, João Marcelo de Castro e Sousa, Ana Amélia de Carvalho Melo-Cavalcante

**Affiliations:** ^1^Laboratory of Experimental Cancerology (LabCancer), Department of Biophysics and Physiology, Federal University of Piauí, Teresina, Brazil; ^2^Postgraduate Program in Pharmaceutical Sciences, Federal University of Piauí, Teresina, Brazil; ^3^Laboratory of Genetic Toxicology (Lapgenic), Department of Biochemistry and Pharmacology, Federal University of Piauí, Teresina, Brazil; ^4^Department of Chemistry, Federal University of Piauí, Teresina, Brazil; ^5^Department of Biology, Federal University of Ceará, Fortaleza, Brazil; ^6^Department of Molecular Biology, Federal University of Paraíba, João Pessoa, Brazil; ^7^Faculty of Medicine, State University of Ceará, Fortaleza, Brazil

## Abstract

*Mimosa caesalpiniifolia* (Fabaceae) is used by Brazilian people to treat hypertension, bronchitis, and skin infections. Herein, we evaluated the antiproliferative action of the dichloromethane fraction from *M. caesalpiniifolia* (DFMC) stem bark on murine tumor cells and the *in vivo* toxicogenetic profile. Initially, the cytotoxic activity of DFMC on primary cultures of Sarcoma 180 (S180) cells by Alamar Blue, trypan, and cytokinesis block micronucleus (CBMN) assays was assessed after 72 h of exposure, followed by the treatment of S180-bearing Swiss mice for 7 days, physiological investigations, and DNA/chromosomal damage. DFMC and betulinic acid revealed similar *in vitro* antiproliferative action on S180 cells and induced a reduction in viable cells, induced a reduction in viable cells and caused the emergence of bridges, buds, and morphological features of apoptosis and necrosis. S180-transplanted mice treated with DFMC (50 and 100 mg/kg/day), a betulinic acid-rich dichloromethane, showed for the first time *in vivo* tumor growth reduction (64.8 and 80.0%) and poorer peri- and intratumor quantities of vessels. Such antiproliferative action was associated with detectible side effects (loss of weight, reduction of spleen, lymphocytopenia, and neutrophilia and increasing of GOT and micronucleus in bone marrow), but preclinical general anticancer properties of the DFMC were not threatened by toxicological effects, and these biomedical discoveries validate the ethnopharmacological reputation of *Mimosa* species as emerging phytotherapy sources of lead molecules.

## 1. Introduction

The history of anticancer drugs is closely related to natural products, since at least 60% of clinical drugs naturally or chemically resemble ones [1]. In this context, Brazil remains at the top of 17 megadiverse countries and the home of around 20% of the world species [2], mainly because approximately 700 new animal species have been discovered each year [3]; it has the greatest number of endemic species on a global scale and about 55,000 plant species (22% of the world total) [4, 5]. Moreover, it is a great producer of medicinal plants for internal consumption as well as for international markets. This invaluable biodiversity encourages biotechnological and pharmacological studies about effective therapy and health recovery [6–8].

A Brazilian dry region named “Caatinga” dominates 7% of the Brazilian territory and is an exclusive biome. It generates particular environmental conditions for steppe climate-adapted flora and fauna and a high number of rare and endemic taxa [9, 10], exhibiting many vegetal families, such as Fabaceae, Anarcadiaceae, Caryocaraceae, Rhamnaceae, Chrysobalanaceae, Clusiaceae, Connaraceae, Sapindaceae, Annonaceae, Combretaceae, and Bignoniaceae [11–15]. Among them, inflorescences from *Mimosa caesalpiniifolia* Benth. (synonym: *Mimosa caesalpiniaefolia*, family Fabaceae), known as “unha de gato,” “sabiá,” and sansão-do-campo,” have been traditionally used by Brazilian people as hedges and windbreaks. Dried fruits and leaves are given as fodder for cattle, goats, and sheep (crude protein ranging from 13.4 to 17.1%) [16] and to treat hypertension [17]. Its bark is popularly used as a coagulant to stop or avoid bleeding and as wound washing to prevent inflammation and skin infections. Moreover, the ingestion of bark infusion alleviates symptoms of bronchitis [16, 18, 19]. Recently, a bioassay-guided phytochemical study found 28 compounds in *M. caesalpiniifolia* leaf extract, and four of them revealed potent antifungal properties against *Candida glabrata* and *Candida krusei* [20]; the latter was often associated with the prior use of azoles and corticosteroids, bone marrow transplantation, malignant hematological diseases, and neutropenia [21].

An expert Brazilian research group about pharmacology of natural products confirmed bioactivity usages for cardiovascular diseases. They reported that ethanolic extracts of different parts of *M. caesalpiniifolia* (leaves, bark, fruit, and inflorescences) cause vasorelaxation, the tea of flowers promotes hypotension and tachycardia, and the ethanolic extract causes hypotension and bradycardia [22]. Based on these ethnopharmacological properties, this work evaluated the antiproliferative action of the dichloromethane fraction from *M. caesalpiniifolia* (DFMC) stem bark on murine tumor cells and the *in vivo* toxicogenetic profile.

## 2. Materials and Methods

### 2.1. Plant Collection and Extract/Fraction Preparation

Plant specimens were collected in May 2010 in Teresina (Piauí, Brazil). A voucher sample (26.824) was deposited at Graziela Barroso Herbarium at Federal University of Piauí (Teresina, Piauí, Brazil). Air-dried plant material was pulverized, extracted with ethanol, concentrated under reduced pressure, and subjected to successive partitioning with dichloromethane as described by Silva et al. [15]. Previously, we isolated betulinic acid [3*β*-hydroxy-lup-20(29)-en-28-oic acid] and verified it as the main compound in the dichloromethane fraction (∼70.3%), as demonstrated by TLC (thin-layer chromatography), GC-qMS (gas chromatograph quadrupole mass spectrometer), HRAPCIMS (high-resolution atmospheric pressure chemical ionization mass spectrometer), ^1^H- and ^13^C-nuclear magnetic resonance, and DEPT analysis [15, 23]. Plant samplings were authorized by the System of Authorization and Information on Biodiversity (SISBIO/BAMA #50090-3) and registered in SisGen (*Sistema Nacional de Gestão do Patrimônio Genético e do Conhecimento Tradicional Associado* #ABC4AC2) according to Brazilian legislation (Federal Law No 13,123/2015). These investigations were performed using the fraction composed of a mixture of molecules because such preparations represent the main folk approach of consumption by the Brazilian population [15].

### 2.2. Animal's Facilities

Adult female Swiss mice (*Mus musculus* Linnaeus, 1758) weighing 20–25 g were obtained from the animal facilities at Universidade Federal do Piauí (UFPI), Teresina, Brazil. All animals were maintained in well-ventilated cages under standard conditions of light (12 h with alternate day and night cycles) and temperature (25 ± 2°C) with free access to food (Nutrilabor™, Campinas, Brazil) and drinkable water. After the tests, mice were euthanized with sodium thiopental (100 mg/kg) (i.p.). All protocols were approved by the Ethical Committee on Animal Experimentation at UFPI (CEUA #034/2014) and followed Brazilian (*Sociedade Brasileira de Ciência em Animais de Laboratório*–SBCAL) and international (Directive 2010/63/EU of the European Parliament and of the Council on the protection of animals used for scientific purposes) rules on the care and use of experimental animals.

### 2.3. *In vitro* Antiproliferative Studies on Sarcoma 180 Cells

#### 2.3.1. *Ex vivo* Cytotoxic Action

Mice with 9 to 10 days of S180 ascitic tumors were euthanized with an overdose of sodium thiopental, and a suspension of S180 cells was taken from the intraperitoneal cavity under aseptic conditions. The cell suspension was centrifuged at 2,000 rpm for 5 min to obtain a pellet, which was washed three times with sterile RPMI medium. The cell concentration was adjusted to 0.5 × 10^6^ cells/mL in RPMI-1640 medium supplemented with 20% fetal bovine serum, 2 mM glutamine, 100 U/mL penicillin, and 100 *μ*g/mL streptomycin (Cultilab™, Brazil), plated in 96-well plates with increasing concentrations (0.8–50 *μ*g/mL) of DFMC and betulinic acid, and incubated at 37°C in a 5% CO_2_ atmosphere (Shel Lab CO_2_ Incubator, USA).

Cell proliferation was assessed by the Alamar Blue™ assay after 72 h. At 48 h of incubation, 20 *μ*L of stock solution (0.156 mg/mL) of Alamar Blue™ (Resazurin, Sigma Aldrich™, USA) was added to each well. Cell proliferation was determined spectrophotometrically using a multiplate reader (T80+ UV/VIS Spectrometer, PG Instruments™, United Kingdom) at 570 and 595 nm. The antiproliferative effect was expressed as the percentage of the control according to Ferreira et al. [12].

#### 2.3.2. Trypan Blue Exclusion Assay

Sarcoma 180 cells (0.5 × 10^6^ cells/mL) plated in 24-well plates were exposed to DFMC at 5, 10, and 25 *μ*g/mL. Doxorubicin (Dox, 0.3 *μ*g/mL) was used as a positive control. Cell viability was examined by the exclusion of trypan blue [24]. Briefly, aliquots of 10 *μ*L were collected from DFMC-treated S180 cultures after 72 h of exposure, and viability was separated into viable blue-marked and nonviable blue-coloured cells in a Neubauer chamber under light microscopy (Biosystems™, USA).

#### 2.3.3. Cytokinesis-Block Micronucleus (CBMN) Assay

Sarcoma 180 cells were plated in 24-well plates (0.5 × 10^6^ cells/mL) and treated with DFMC at 5, 25, and 50 *μ*g/mL. After 44 h at 37°C, cytochalasin B (Sigma Aldrich, USA, 6 *μ*g/mL) was added, and the cells were reincubated for an additional 28 h. At 72 h, the cultures were transferred to tubes and centrifuged at 800 rpm for 5 minutes. Then, the supernatant was removed, and the body of the cell bottom was enlarged and centrifuged again before the addition of 2 mL of fixing solution (methanol and acetic acid, ratio 5 : 1) and 3 drops of formaldehyde 37% (Vetec™, Brazil). This procedure was repeated 3x using fixing solution 3 : 1 without formaldehyde. Supernatants were discarded, and 2-3 drops of cell suspension were dripped onto slides and stained with Giemsa for 5 min [25]. Considering blind examination, a total of 2000 cells by concentration were counted by optical microscopy at 1000x (1000 cells/slide) to count buds, bridges, and micronuclei.

### 2.4. *In vivo* Assays

#### 2.4.1. Assessment of Antitumor Capacity, Physiological Parameters, and Histological Aspects

Ten-day-old S180 ascites tumor cells were removed from the peritoneal cavity, counted (6 × 10^6^ cells/mL) and subcutaneously implanted into the right hind axillary of healthy Swiss animals. On the next day, they were randomly divided into four groups (*n* = 10 each). DFMC dissolved in dimethylsulfoxide (DMSO 5%, Vetec™, Brazil) was intraperitoneally injected at 50 or 100 mg/kg/day for 7 days. Negative and positive controls received DMSO 5% and 5-fluoruracil (5-FU, 25 mg/kg/day, Sigma Aldrich™, USA), respectively [26].

All animals were anaesthetized on day 8 with ketamine (90 mg/kg)-xylazine (4.5 mg/kg) for cardiac puncture blood collection [27] using sterile tubes and heparinize pipettes to determine hematological parameters (erythrocytes, leukocytes, platelets, hemoglobin, and hematocrit) in peripheral blood samples using an automatic analyzer of hematologic cells (SDH-3 Vet Labtest™, Brazil). The absolute count of white cellular subtypes was calculated as the product of its respective differential percentage and total leukocyte count. For biochemical analysis, blood samples were centrifuged at 2,000 rpm for 5 minutes. Physiological markers of the liver [blood urea nitrogen (BUN), glutamate oxaloacetate transaminase (GOT), glutamate pyruvate transaminase (GPT), alkaline phosphatase (ALP)] and kidneys (creatinine) were evaluated according to Labmax Plenno Labtest™. Subsequently, all animals were euthanized to dissect out the liver, kidneys, spleen, stomach, heart, and lungs to estimate wet relative weights and for macroscopic analysis. Next, organs were fixed with 10% buffered formalin, processed, and cut into small pieces to prepare histological sections (4–7 *μ*m). Staining was carried out with hematoxylin and eosin (H&E, Vetec™, Brazil). Morphological blind analyses were performed under light microscopy (Olympus™, Japan) by an expert pathologist.

#### 2.4.2. Determination of Chromosomal Damages

The femurs were removed and carefully cleaned, and proximal epiphyses were sectioned. Bone marrow samples were collected using 5 mL syringes filled with 0.5 mL of sterile fetal bovine serum (Cultilab™, Brazil), centrifuged, and homogenized. Drops of cell suspension were transferred to slides to prepare smears (two slides/animal), fixed and stained by the Leishman method. All analyses were blindly performed under light microscopy (Olympus™, Japan) with magnifications of 200x and 400x. We considered micronuclei to be rounded structures, with a diameter of 1/5 to 1/20 found in young erythrocytes and identified by bluish staining. A total of 1,000 polychromatic erythrocytes (PCEs) was quantified per slide (two slides/animal) [28–30].

### 2.5. Statistical Analysis

Half maximal inhibitory concentration (IC_50_) and their 95% confidence intervals were calculated by nonlinear regression (GraphPad Prisma 9.0, Intuitive Software for Science, USA). Statistical differences were evaluated comparing data [mean ± standard error of mean (S.E.M.)] by one-way analysis of variance (ANOVA) and Newman–Keuls test as *post hoc* test (*p* < 0.05). All *in vitro* studies were carried out in duplicate (*n* = 3/concentration) and represent independent biological evaluations.

## 3. Results

### 3.1. *In vitro* Antiproliferative Action on Sarcoma 180 Cells: Cytotoxicity, Chromosomal Changes, and Cell Death Pattern

**Figure 1:**
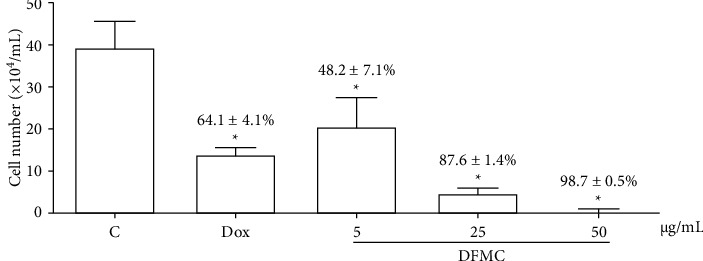
The cell number of viable cells was determined by trypan blue staining and analyzed by light microscopy after 72 h of exposure to the dichloromethane fraction from *Mimosa caesalpiniifolia* (DFMC) stem bark. The percentage of viability reduction in relation to the negative control is described above. The negative control (C) was treated with the vehicle used to dilute the tested substance. Doxorubicin (Dox, 0.3 *μ*g/mL) was used as a positive control. The results are expressed as mean ± standard error of measurement (S.E.M.) from two independent experiments. ^*∗*^*p* < 0.05 compared to the control by ANOVA followed by student Newman–Keuls test.

DFMC and betulinic acid revealed similar *in vitro* antiproliferative activity against S180 cells after 72 h of incubation, with IC_50_ values of 29.0 (24.9–33.6) *μ*g/mL and 33.7 (30.1–37.6) *μ*g/mL, respectively (*p* > 0.05, Table 1). Afterwards, this action was confirmed by trypan blue assay (Figure 1), a direct method to detect cytotoxicity, which showed that all concentrations of DFMC (5, 25, and 50 *μ*g/mL) reduced the number of viable cells (48.2 ± 7.1, 87.6 ± 1.4, and 98.7 ± 0.5%, respectively) when compared to the negative control (*p* < 0.05).

**Figure 2:**
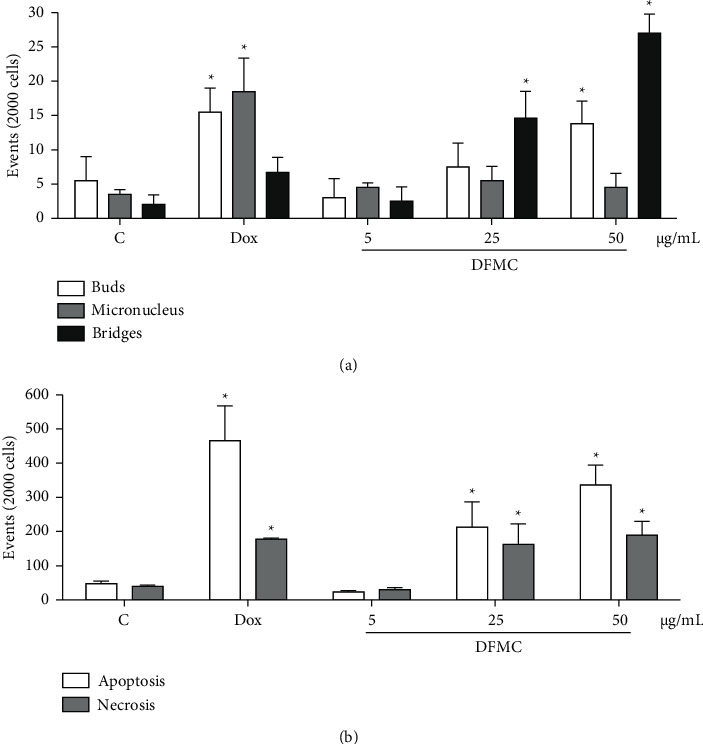
*Ex vivo* chromosomal changes and cell death pattern in sarcoma 180 cells determined by micronucleus assay with cytokinesis block after 72 h exposure to the dichloromethane fraction from *Mimosa caesalpiniifolia* (DFMC) stem bark. The negative control (C) was treated with the vehicle used to dilute the tested substance (DMSO 0.1%). Doxorubicin (Dox, 0.3 *μ*g/mL) was used as a positive control. The results are expressed as mean ± standard error of measurement (S.E.M.) from two independent experiments. ^*∗*^*p* < 0.05 compared to the control by ANOVA followed by student Newman–Keuls test.

Morphological analysis of DFMC-treated Sarcoma 180 cells did not show significant micronucleus induction (4.5 ± 0.7, 5.5 ± 2.1, and 4.5 ± 2.1 for 5, 25, and 50 *μ*g/mL, respectively) in relation to the negative control (3.5 ± 0.7, *p* > 0.05, Figure 2(a)). On the other hand, bridges (14.6 ± 3.9 and 27.0 ± (2) and buds (13.8 ± 3.3) were observed at 25 and 50 *μ*g/mL and 50 *μ*g/mL (*p* < 0.05) when compared to the negative control (2.0 ± 1.4 and 5.5 ± 3.5), respectively. Such chromosomal damage was corroborated by morphological features of apoptosis (213.0 ± 73.5 and 337.0 ± 57.9) and necrosis (162.5 ± 60.1 and 189.5 ± 40.3) at 25 and 50 *μ*g/mL (*p* < 0.05, Figure 2(b)) in the presence of cell rarefaction and vacuolization. As expected, Dox increased buds (15.5 ± 3.5) and micronuclei (18.5 ± 4.9) and caused typical findings of apoptosis (466.0 ± 101.8) and necrosis (177.5 ± 3.5) (*p* < 0.05).

### 3.2. *In vivo* Antitumoral Activity

Experimentally transplanted mice with Sarcoma 180 cells and treated with DFMC (50 and 100 mg/kg/day) for 7 days revealed a significant reduction in tumor growth [(0.28 ± 0.04 g (64.8 ± 5.3%) and 0.16 ± 0.07 g (80.0 ± 8.4%)] when compared to the negative control (0.80 ± 0.13 g, respectively). Tumor reduction was also noted in the positive control group treated with 5-FU [0.11 ± 0.03 g (82.8 ± 4.2%)] (*p* < 0.05, Table 2).

**Figure 3:**
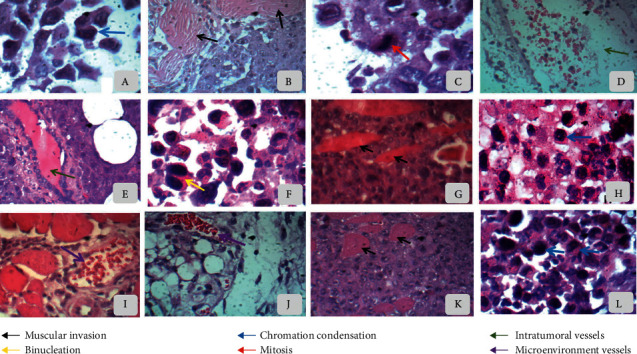
Morphology of sarcoma 180 tumor cells from swiss mice after 7 days of treatment with dichloromethane fraction from *Mimosa caesalpiniifolia* stem bark. Animals were treated by intraperitoneal injection (50 mg/kg/day: g, h, and i; 100 mg/kg/day: j, k, and l). The negative control was treated with the vehicle used to dilute the substance (DMSO 5%: a–d). 5-Fluorouracil was used as a positive control (e and f). Hematoxylin-eosin staining. Light microscopy magnification, 100x-400x.

The negative control group showed characteristics of malignant neoplasms consisting of round and polyhedral cells, anisocariosis, binucleation, mitoses, and different degrees of cell and nuclear pleomorphism, chromatin condensation, and extensive areas of muscle invasion (Figures 3(a)–3(d)). Tumor samples from 5-FU 25 mg/kg/day and FDCM 50 and 100 mg/kg/day also revealed the typical morphology of neoplastic cells, although rare mitoses were observed, which indicated a reduction in proliferation (Figures 3(e)–3(l)). 5-FU-treated tumors showed larger blood vessels and well vascularized sarcomas, similar to those noted in negative control tumors (Figure 3(e)). On the other hand, DFMC-treated Sarcoma 180 tumors treated with 50 and 100 mg/kg/day exhibited poorer peri- and intratumor quantities of vessels. In such tumors, vascularization was partially restricted to the adipose tissue surrounding the tumor (Figures 3(i)–3(j)).

### 3.3. Physiological Parameters

In the next step, we assessed macroscopic and microscopic parameters of key organs and the hematological profile of Sarcoma 180-bearing mice after treatment with DFMC. First, we found a reduction in body weight gain in DFMC-treated animals (20.6 ± 0.8 and 21.4 ± 1.6 g, for 50 and 1000 mg/kg/day) in a similar way to the 5-FU group (20.1 ± 0.9 g) when compared to the negative control (26.3 ± 2.2 g, *p* < 0.05, Table 1). Wet relative weight reduction of spleens was noted in both doses of DFMC (0.2 ± 0.08 and 0.2 ± 0.03 g/100 g of body weight) and in 5-FU-treated animals (0.2 ± 0.04 g), but liver decrease was observed in 5-FU-treated animals only (4.7 ± 0.1 g) in comparison with the negative group (0.4 ± 0.04 g and 6.0 ± 0.4 g, respectively, *p* < 0.05).

Hematological analysis of DFMC-treated animals showed neutrophilia (33.8 ± 3.2%), lymphocytopenia (61.5 ± 3.6%), a reduction in eosinophils (0.4 ± 0.2%), and a slight increase in GOT levels (315.3 ± 8.9 U/mL) (*p* < 0.05, Table 3). Animals exposed to 5-FU showed intense leukopenia (1.6 ± 0.3/mm^3^) due to declines in neutrophils (12.9 ± 1.3%), monocytes (0.6 ± 0.2%) and eosinophils (0.6 ± 0.3%) compared to the animals from the negative group (5.1 ± 0.4/mm^3^, 18.8 ± 2.8%, 1.8 ± 0.3% and 1.8 ± 0.4%, respectively, *p* < 0.05).

### 3.4. Histological Alterations

**Figure 4:**
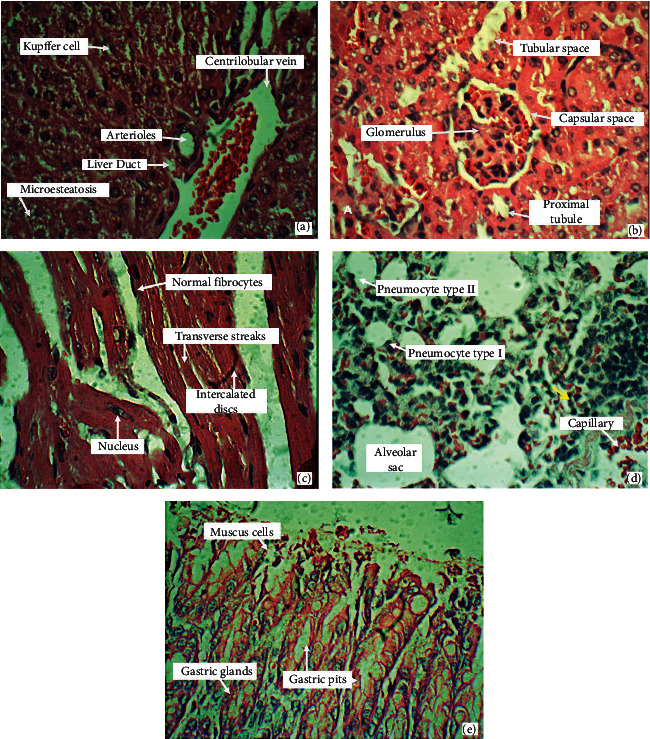
General morphology of livers (a), kidneys (b), hearts (c), lungs (d), and stomachs (e) from Swiss mice after 7 days of treatment with dichloromethane fraction from *Mimosa caesalpiniifolia* stem bark (50 or 100 mg/kg/day) or vehicle used to dilute the substance (DMSO 5%). Important changes among these groups were not observed. Hematoxylin-eosin staining. Light microscopy magnification, 400x.

**Figure 5:**
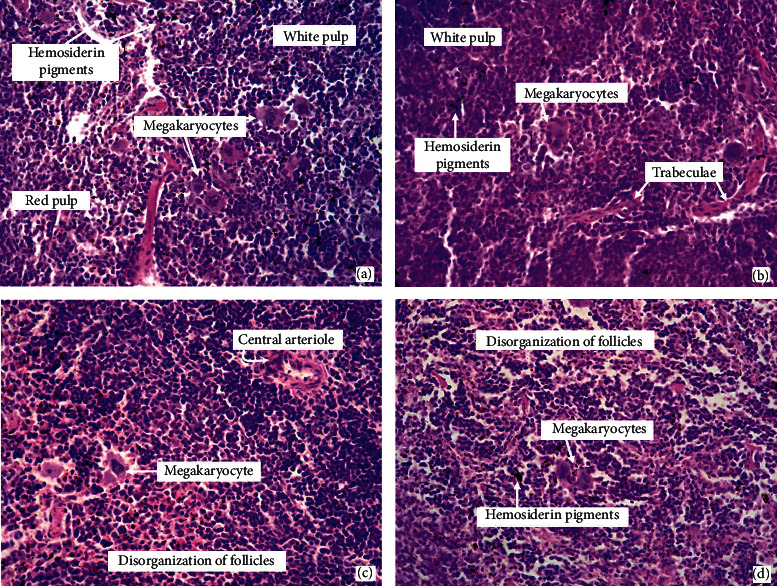
Spleen morphology of Swiss mice after 7 days of treatment with dichloromethane fraction from *Mimosa caesalpiniifolia* stem bark (50 mg/kg/day (c); 100 mg/kg/day (d)), vehicle used to dilute the substance DMSO 5% (a) or 5-fluorouracil 25 mg/kg/day (b). Hematoxylin-eosin staining. Light microscopy magnification, 400x.

Animals from the negative control group and treated with DFMC (50 and 100 mg/kg/day) did not show signs of toxicity, with similarity among organs from these groups. Livers did not exhibit hyperplasia, hemosiderin pigments, infiltration of leukocytes, cell swelling, portal congestion, or areas of necrosis, although microesteatosis was detected in all groups (Figure 4(a)). Kidneys present no swelling, tubular degeneration, vascular congestion, or necrosis focus (Figure 4(b)); in hearts, there were no areas of degeneration or fibroblasts proliferation and striations were clearly visible (Figure 4(c)); lungs showed bronchioles and visible alveolar spaces, absence of mono and polymorphonuclear cells or areas of necrosis (Figure 4(d)); stomachs showed normal mucosa and submucosa, absence of hemorrhagic streaks, a cardiac region with a keratinized squamous lining, no changes in chorion and easy visualization of parietal and main cells (Figure 4(e)). Spleens showed megakaryocytes and hemosiderin pigments in all groups. Disorganization of lymphoid follicles and relative reduction of the white pulp were observed in the 5-FU (Figure 5(b)) and DFMC-treated animals (Figures 5(c) and 5(d)). On the other hand, 5-FU-treated animals showed slight hepatocyte swelling and suggestion of mild changes in fatty metabolism since macroesteatosis was noted, and kidneys presented swelling of tubular cells and foci of atrophic glomeruli (results not shown).

### 3.5. *In vivo* Chromosomal Damage

DFMC increased micronucleated polychromatic erythrocytes in the bone marrow of mice in a dose-dependent manner (50 and 100 mg/kg/day: 11.5 ± 0.2 and 26.0 ± 2.1, respectively) compared to the vehicle group (2.8 ± 0.2, *p* < 0.05). As expected, 25 mg/kg/day 5-FU caused clastogenic effects (14.0 ± 0.1, *p* < 0.05).

## 4. Discussion

In the last century, the development of cytotoxic agents has revolutionized anticancer therapy. Adjuvant treatments with antiproliferative substances have demonstrated an indisputable advantage when compared to traditional treatments based on surgery and monochemotherapy, making it possible to cure neoplasms such as acute childhood leukemia, Hodgkin and non-Hodgkin's lymphomas, and germ cell tumors [31, 32]. However, the great heterogeneity of tumor cells makes treatment difficult and facilitates the manifestation of resistance [33], which stimulates the search for new chemotherapeutic agents.

Initially, the antiproliferative action of DFMC was evaluated in primary cultures of Sarcoma 180 cells. *In vitro* cytotoxicity tests in cell cultures are important for the evaluation of antitumor agents, and at least during the screening phase, they have reduced *in vivo* tests on animals. In addition, they are widely used as alternative methods to pharmacological tests on isolated organs [26, 34]. Herein, DFMC and its majority compound betulinic acid revealed similar cytotoxic capacity on S180 cells by Alamar blue assay, whose action was confirmed by cell viability reduction in trypan blue exclusion tests. Some reports, including the American National Cancer Institute (NCI-USA) [35], suggest that IC_50_ values around 30 *μ*g/mL are a suitable outcome to consider extracts and fractions promising substances for further purification and biological studies [12, 15]. Recently, we reported that DFMC has higher cytotoxic action against different types of tumor tissues (promyelocytic leukemia, HL-60; glioblastoma, SF-295; ovarian, OVCAR-8; colon, HCT-116) than hexane and water extracts. DFMC did not produce hemolysis and showed higher potential as a cytotoxic agent than betulinic acid for the SF-295 and HL-60 lines [20, 36], corroborating the findings described here for S180 cells.

Phytochemical investigation of extracts from *Mimosa* species revealed the existence of terpenes, flavonoids, steroids, phenols (especially tannins), and fatty acid derivatives in different parts of the plant (leaves, fruits, flowers, branches, and stem bark) [36–40], mainly betulinic acid, lupeol, phytol, lactic acid, *α*-tocopherol, stigmasterol, *β*-sitosterol, sitostenone, and stigmasta-4,22-dien-3-one, which had been identified in dichloromethane, ethanolic, and hexane fractions of leaves and barks from *M. caesalpiniifolia* [15, 36, 40], suggesting that the antiproliferative potential of DFMC may be attributed, at least in part, to its chemopreventive action. In this context, Silva et al. [15] stated the scavenger activity of *M. caesalpiniifolia* extracts, whose presence of phenolic compounds was confirmed by ultraviolet-visible spectroscopy and thin-layer chromatography.

Betulinic acid, a naturally occurring pentacyclic triterpenoid, is the main compound in the fraction (∼70.3%) [15, 23], and both samples (DFMC and isolated molecule) have similar bioactivity on S180 cells (*p* > 0.05), confirming reports about the antiproliferative action of betulinic acid in many types of cancers [41–50].

To complement the *ex vivo* cytotoxic analysis on S180 tumor cells and *in vivo* pharmacological safety, cytokinesis-block micronucleus (CBMN) assays were performed to measure micronuclei quantification and DNA damage in mammalian cell cultures [28]. Apart from the evaluation of micronuclei, the CBMN cytome assay allows the assessment of other relevant biodosimetric markers: nucleoplasmic bridges, nuclear buds, proportion of dividing cells (parameter of cytostasis), and cells undergoing apoptosis and necrosis (parameters of cytotoxicity). Therefore, this technique was updated to detect chromosomal breaks, DNA rearrangements, chromosomal losses, cytostasis, and to separate types of cell death [25, 28, 51, 52]. Therefore, for the first time, an increase in chromosomal damage represented by (i) nucleoplasm bridges: a biomarker of dicentric chromosomes, resulting from the fusion of the final telomeres after DNA double-strand breaks or DNA misrepair/rearrangements; (ii) buds: a biomarker of gene amplification and originating from interstitial or terminal acentric fragments; and (iii) morphological features of apoptosis and necrosis in S180 cells at higher concentrations of DFMC was noted. Meanwhile, both doses of DFMC also induced the emergence of micronucleated polychromatic erythrocytes in bone marrow. Previously, Silva et al. [23] reported an ethanolic extract from *M. caesalpiniifolia* leaves with maximum cytotoxicity on breast carcinoma MCF-7 cells at 320 *μ*g/mL and morphological changes suggestive of apoptosis, including DNA fragmentation and nuclear chromatin condensation.

Recently, we also showed that micronuclei formation and changes indicating mutagenic index in DFMC-treated roots were not detected, although this fraction has inhibited growth of *Allium cepa* roots and increase amount of bridges in dividing meristematic cells, which indicates capacity for mitotic index reduction as seen as dropping of cells at metaphase, anaphase, and telophase phases and cycle arrest at prophase [15]. Regardless, it is likely that DNA/chromosomal damage is a sign of nonselective mechanism(s) in tumor or normal dividing cells. Therefore, *in vitro* (bridges and buds) and *in vivo* (micronucleus) clastogenic findings led to cell cycle arrest as a “cellular escape” from death, mainly if we consider the antiproliferative action of DFMC on human normal leukocytes well [15].

Indeed, antineoplastic agents induce DNA strand breaks in mammalian cells, as seen with inhibitors of topoisomerase I (camptothecin) and topoisomerase II (etoposide) [53] and 5-FU. 5-FU is a widely used antimetabolite to treat breast adenocarcinomas and cancers of the gastrointestinal tract and head and neck due to its inhibitory action on the enzyme thymidylate synthase [54], among other mechanisms, despite its unblemished *in vivo* clastogenic activity [55]. However, genotoxicity does not mean mutagenicity because some genome injuries are biochemically fixed, which indicates that antineoplastic acute toxic consequences (e.g., inhibition of growth and cell division) are not automatically linked to chromosomal loss/impairments [56].

The cytotoxic activity on cancer cells using *in vitro* models may not reflect *in vivo* findings, since the latter considers pharmacokinetic and pharmacodynamic variables, such as ligand binding to specific receptors, downstream cascade, involvement of second messengers, water/lipid solubility, bioavailability, first-pass metabolism, and renal excretion [57, 58]. Therefore, combining these two types of scientific tools is appropriate for a more complete assessment of a substance with antiproliferative action. For the first time, the amazing antitumor action of a dichloromethane fraction from *M. caesalpiniifolia* stem bark on *in vivo* proliferating Sarcoma 180 cells was demonstrated. *In vivo* studies have already shown that betulinic acid inhibits the growth of human ovarian IGROV-1 carcinoma xenographic tumors at 100 mg/kg/day and increases the survival rate of mice [46].

No specific changes were noted during histopathological analysis of the Sarcoma 180 tumors [34], but it is important to emphasize that local vascularization from DFMC-treated animals was predominantly confined to the adipose tissue surrounding the tumors. These unexpected findings were not described before and suggest that the fraction interferes with the cell cycle of Sarcoma 180 cells and inhibits angiogenesis, which obviously alters the stromal environment, such as the local pH, partial pressure of oxygen and carbon dioxide, input of nutrients/growth factors, and cleaning of metabolic residues [57], all essential primary conditions for cellular division and tumor growth. Molecular studies are underway to confirm such antiangiogenic potential. These data corroborate our findings about the biomedical potential of *M. caesalpiniifolia* and inspired us to assess the pharmacological safety profile of the fraction, taking into consideration its promising phytotherapy properties.

The development of new (phyto)pharmaceutical products includes not only pharmacodynamic discoveries but also essential data about the pharmacokinetics profile, therapeutic window, and pharmacological safety, including systemic and genetic toxicology [58, 59]. These assessments allow the exclusion of undesirable drug candidates and save time, material and human resources. In the case of plant toxicity/poisoning, its harmful action must be proven experimentally. For humans, this experimental reproduction should be carried out in the same animal species, naturally affected, or related species (e.g., mice and rats), although different susceptibilities to the effects of toxic herbals among species are a common mark [60, 61].

Acute signs of systemic toxicity are loss of body mass and expansion or involution of key organs in mammals exposed to an investigational drug [62]. Weight loss is one of the most common side effects after chemotherapy cycles with 5-FU or doxorubicin, since the gastrointestinal system is one of the main nonspecific targets of nontarget antiproliferative agents, causing seasickness, suppression of appetite, vomiting, and diarrhea [63]. Loss of body weight and reduction of spleens were macroscopic manifestations found in the 5-FU- and DFMC-treated groups, but signs of diarrhea were not seen in the DFMC-treated groups. Spleen diminution is another very common side effect found in S180-bearing mice under experimental treatment with promising antitumoral substances [26, 64] and reflects lymphocytopenia seen in 5-FU- and DFMC-treated groups and strong leukopenia in 5-FU-treated mice, which was confirmed by disorganization of lymphoid follicles and size reduction of white pulps.

*In vivo* toxicological studies with DFMC were not found in the literature, but oral subacute treatment of rats for 32 days with 750 mg/kg/day ethanolic extract from *M. caesalpiniifolia* leaves caused weight loss, hepatomegaly, and an increase in adrenal and pituitary glands [40], but serum biochemical evaluation (alkaline phosphatase, GOT, urea, and creatinine) did not identify renal or liver changes. On the other hand, we noted that the 100 mg/kg/day DFMC-treated group revealed a slight but significant increase in GOT.

Transaminases (GOT and GTP) are found in all human systems and many organs, but they are more present in the cytoplasm (100% GTP/20% GOT) or mitochondria (80% GOT) of hepatocytes, since they catalyze transamination reactions, working central providers of secondary metabolites to the citric acid cycle. Any type of liver injury may sensibly increase serum GTP concentrations, a classic biomarker to assess acute or chronic hepatic damage, but its origin can have kidney, heart, or muscle reasons because these organs also possess higher GTP concentrations in comparison with other tissues [65]. On the other hand, GOT is more abundant in heart, skeletal muscle, kidneys, brain, and red blood cells [66], with lower concentrations in skeletal muscle and kidney. Although GTP is more specific for detecting liver damage, ischemic or toxic damage to zone 3 of the hepatic acinus may change GOT levels since this region has greater GOT concentrations [65].

Histological changes were not found in livers from DFMC-treated animals. Thus, it is probable that higher levels of GOT may be associated with muscle damage and/or trauma after continual intraperitoneal injections because this procedure can result in aminotransferase release, and an increase in GOT is common in such situations [66].

**Figure 6:**
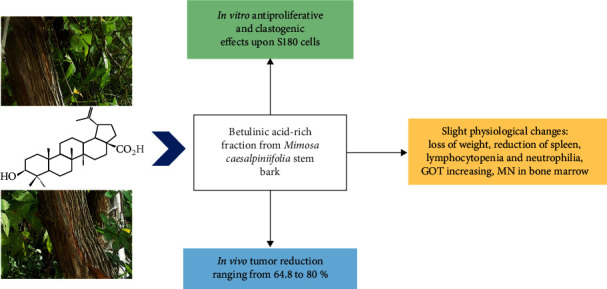
Summary of the antiproliferative, genotoxic, antitumoral, and toxicological effects of a betulinic acid-rich fraction from *Mimosa caesalpiniifolia* stem bark.

The majority of clinically available anticancer medications provoke strong side effects, especially suppression of bone marrow and immune response, toxicity on hepatocytes, cardiac myocytes and enterocytes, mucositis, weight and hair loss (incidence of 65%), opportunistic infections, seasickness, vomiting, chemotherapy-related anorexia, peripheral neuropatia, and tiredness [33, 63, 67–69], whose types and intensity depend on the mechanism(s) of action and idiosyncratic reactions. Based on nonsevere organic findings, we believe that the preclinical general anticancer properties of DFMC are not threatened by toxicological effects (Figure 6).

## 5. Conclusions

A betulinic acid-rich fraction from *Mimosa caesalpiniifolia* stem bark showed, for the first time, *in vitro* and *in vivo* antiproliferative capacity on Sarcoma 180 tumors and induction of nonselective chromosomal damage (bridges, buds, and micronucleus) to dividing murine cells. Such antimitotic action was associated with detectible physiological changes, indicating side effects (loss of weight, reduction of spleen, lymphocytopenia, and neutrophilia and increasing of GOT and micronucleus in bone marrow). These biomedical discoveries validate the ethnopharmacological reputation of *Mimosa* species as emerging phytotherapy sources of lead molecules.

## Figures and Tables

**Table 1:** Cytotoxic activity of the dichloromethane fraction and betulinic acid from *Mimosa caesalpiniifolia* (DFMC) stem bark on primary culture of sarcoma 180 cells after 72 h of exposure evaluated by alamar blue assay.

Sample	IC_50_ (*μ*g/mL)	
	Sarcoma 180 cells	*R* ^2^
DFMC	29.0 (24.9–33.6)	0.9278
Betulinic acid	33.7 (30.1–37.6)	0.9292
Doxorubicin	1.9 (1.4–2.4)	0.9801

Data are presented as IC_50_ values and 95% confidence intervals. Doxorubicin was used as positive control. Experiments were performed in duplicate.

**Table 2:** Effect of the dichloromethane fraction from *Mimosa caesalpiniifolia* (DFMC) stem bark on the relative weight of key organs and on the tumor growth of sarcoma 180-bearing swiss mice after 7 days of intraperitoneal treatment.

Substance	Dose (mg/kg/day)	Mice weight (g)	Liver	Kidney	Spleen	Stomach	Lungs	Tumor (g)	Tumor inhibition (%)
			g/100 g body weight						
Negative control	—	26.3 ± 2.2	6.0 ± 0.4	1.1 ± 0.1	0.4 ± 0.04	1.0 ± 0.1	0.8 ± 0.1	0.80 ± 0.13	—
5-FU	25	20.1 ± 0.9^*∗*^	4.7 ± 0.1^*∗*^	1.2 ± 0.1	0.2 ± 0.04^*∗*^	1.1 ± 0.1	0.8 ± 0.1	0.11 ± 0.03^*∗*^	82.8 ± 4.2^*∗*^

DFMC	50	20.6 ± 0.8^*∗*^	5.8 ± 0.2	1.2 ± 0.1	0.2 ± 0.08^*∗*^	1.1 ± 0.1	1.0 ± 0.2	0.28 ± 0.04^*∗*^	64.8 ± 5.3^*∗*^
	100	21.4 ± 1.0^*∗*^	5.9 ± 0.2	1.3 ± 0.1	0.2 ± 0.03^*∗*^	1.2 ± 0.5	0.8 ± 0.1	0.16 ± 0.07^*∗*^	80.0 ± 8.4^*∗*^

Values are means ± S.E.M. (*n* = 10 animals/group). The negative control was treated with the vehicle used to dilute the drug (DMSO 5%). 5-Fluorouracil (5-FU) was used as positive control. ^*∗*^*p* < 0.05 compared with the negative control by ANOVA followed by Newman–Keuls test.

**Table 3:** Hematological and biochemical parameters of mice intraperitoneally treated with dichloromethane fraction from *Mimosa caesalpiniifolia* stem bark for 7 days.

Parameters	Negative control	5-FU 25 mg/kg/day	Dichloromethane fraction from *Mimosa caesalpiniifolia*	
			50 mg/kg/day	100 mg/kg/day
Erythrocytes (mm^3^)	4.5 ± 0.2	4.4 ± 0.2	5.0 ± 0.1	4.9 ± 0.2
Hemoglobin (g/dL)	13.6 ± 0.7	13.3 ± 0.8	15.4 ± 0.4	15.0 ± 0.7
Hematocrit (%)	40.7 ± 2.3	40.1 ± 2.3	46.3 ± 1.1	44.9 ± 2.2
VCM (fL)	90.8 ± 0.5	90.6 ± 0.6	91.8 ± 0.3	91.6 ± 0.5
HCM (pg)	30.2 ± 0.2	30.2 ± 0.2	30.5 ± 0.1	30.5 ± 0.2
CHCM (g/dL)	33.3 ± 0.1	33.1 ± 0.1	33.2 ± 0.1	33.3 ± 0.1
Platelets (mm^3^)	3.6 ± 0.5	2.9 ± 0.2	3.4 ± 0.2	3.4 ± 0.2
Total leukocytes (mm^3^)	5.1 ± 0.4	1.6 ± 0.3^∗^	5.4 ± 0.6	4.9 ± 0.7
Neutrophils (%)	18.8 ± 2.8	12.9 ± 1.3^∗^	23.3 ± 4.1	33.8 ± 3.2^∗^
Rods (%)	0.4 ± 0.2	0.4 ± 0.3	0.6 ± 0.2	1.8 ± 0.7
Lymphocytes (%)	77.3 ± 3.0	85.6 ± 1.8	73.7 ± 4.1	61.5 ± 3.6^∗^
Monocytes (%)	1.8 ± 0.3	0.6 ± 0.2^∗^	1.7 ± 0.6	2.6 ± 0.8
Eosinophils (%)	1.8 ± 0.4	0.6 ± 0.3^∗^	0.7 ± 0.3^∗^	0.4 ± 0.2^∗^
Basophils (%)	0.0	0.0	0.0	0.0
GOT (U/mL)	286.9 ± 5.8	303.2 ± 7.6	280.8 ± 9.1	315.3 ± 8.9^∗^
GTP (U/mL)	158.8 ± 2.6	157.5 ± 4.3	161.6 ± 5.0	156.3 ± 1.1
ALP (U/L)	112.3 ± 5.5	131.2 ± 9.8	101.2 ± 3.4	93.8 ± 6.6
Creatinine (mg/dL)	0.5 ± 0.05	0.5 ± 0.08	0.4 ± 0.01	0.4 ± 0.04
BUN (mg/dL)	48.9 ± 4.2	37.7 ± 2.4	41.3 ± 6.6	42.8 ± 3.1

MCH, mean corpuscular hemoglobin; MCV, mean corpuscular volume; MCHC, mean corpuscular hemoglobin concentration; BUN, blood urea nitrogen; GOT, glutamate oxaloacetate transaminase; GPT, glutamate pyruvate transaminase; ALP, alkaline phosphatase. Values are means ± S.E.M. (*n* = 10 animals/group). The negative control was treated with the vehicle used to dilute the drug (DMSO 5%). 5-Fluorouracil (5-FU) was used as positive control. ^∗^*P* < 0.05 compared with the negative control by ANOVA followed by Newman–Keuls test.

## Data Availability

The data sets used and/or analyzed during the present study are available from the corresponding author on reasonable request.
